# Spatial updating in virtual reality for reproducing object locations in vista space—Boundaries, landmarks, and idiothetic cues

**DOI:** 10.3389/fpsyg.2023.1144861

**Published:** 2023-06-22

**Authors:** Zhanna Borodaeva, Sven Winkler, Jennifer Brade, Philipp Klimant, Georg Jahn

**Affiliations:** ^1^Applied Geropsychology and Cognition, Institute of Psychology, Faculty of Behavioural and Social Sciences, Chemnitz University of Technology, Chemnitz, Germany; ^2^Production Systems and Processes, Institute for Machine Tools and Production Processes, Faculty of Mechanical Engineering, Chemnitz University of Technology, Chemnitz, Germany

**Keywords:** spatial updating, spatial memory, virtual reality, landmark, boundary, translation, walking, pointing

## Abstract

Keeping track of locations across self-motion is possible by continuously updating spatial representations or by encoding and later instantaneously retrieving spatial representations. In virtual reality (VR), sensory cues to self-motion used in continuous updating are typically reduced. In passive translation compared to real walking in VR, optic flow is available but body-based (idiothetic) cues are missing. With both kinds of translation, boundaries and landmarks as static visual cues can be used for instantaneous updating. In two experiments, we let participants encode two target locations, one of which had to be reproduced by pointing after forward translation in immersive VR (HMD). We increased sensory cues to self-motion in comparison to passive translation either by strengthening optic flow or by real walking. Furthermore, we varied static visual cues in the form of boundaries and landmarks inside boundaries. Increased optic flow and real walking did not reliably increase performance suggesting that optic flow even in a sparse environment was sufficient for continuous updating or that merely instantaneous updating took place. Boundaries and landmarks, however, did support performance as quantified by decreased bias and increased precision, particularly if they were close to or even enclosed target locations. Thus, enriched spatial context is a viable method to support spatial updating in VR and synthetic environments (teleoperation). Spatial context does not only provide a static visual reference in offline updating and continuous allocentric self-location updating but also, according to recent neuroscientific evidence on egocentric bearing cells, contributes to continuous egocentric location updating as well.

## 1. Introduction

While moving through an environment, the own location and orientation within the environment as well as attended egocentric location representations are effortlessly updated. For instance, imagine yourself walking on a sidewalk and having noticed a small box 20 m ahead on the other side of the street. If a stopping bus occluded the box and you continued walking, you could still point to the box. If the box would be gone after the bus had moved on, you could point to the former location of the box from your novel standpoint because the egocentric memory representation of the box location needed for pointing has been updated while you walked forward. Such updating happens effortlessly for a low number of object locations when walking in the real world (e.g., Attneave and Farrar, 1977). In virtual reality (VR), however, cues about self-motion that are processed in spatial updating are reduced. In the experiments reported here, we studied strengthened optic flow and idiothetic (body-based) cues potentially supporting spatial updating of encoded locations across forward self-motion in VR. In addition to dynamic cues about self-motion, we also varied static visual cues that as spatial reference can serve self-localization and thus egocentric object-location updating as well as allocentric encoding and reproducing of object locations. If the box on the sidewalk had been located in front of a store window or close to a fire hydrant, these elements of spatial context would have provided a reference in encoding and had supported reproducing the box location. We quantify such supporting effects of boundaries and landmarks on spatial memory across forward self-motion in VR as decreased bias and increased precision.

## 2. Literature review and research questions

### 2.1. Continuous and instantaneous updating

Imagine a sparse environment, say a sand surface in a desert. You throw a coin ahead of you and then a second one. They disappear in the sand. After a few steps forward, you can still point to the coins. Their egocentric representations have been continuously updated. If you had walked forward with your eyes closed, this would have happened by dynamic, non-visual, and body-based (idiothetic) cues alone. Continuous updating of the ego-location while walking is commonly called path integration and enables one to point to egocentrically encoded locations after self-motion. With open eyes, dynamic visual information (optic flow) contributes to the continuous updating of the ego-location and thus also to the continuous updating of egocentric location representations. In an environment that contains boundaries and landmarks, optic flow is strengthened and continuous updating of the ego-location is supported by the spatial reference that they provide both for self-localization in egocentric as well as allocentric representations of the environment.

With spatial reference (i.e., in an environment that is not sparse), both the ego-location in an environment and previously encoded but now invisible target locations can be determined after self-motion by instantaneous updating. Particularly, if a target location is close to a boundary or landmark, the egocentric representation required for pointing to the target location can be constructed after self-motion instantaneously. For example, imagine that one of the coins had disappeared in the sand close to a rock. The rock and thus the invisible target location are obvious after self-motion, and instantaneous updating for pointing is trivial.

The two ways of retrieving locations after self-motion—based on continuous and instantaneous updating (von der Heyde and Riecke, 2002)—are both possible to employ but the degree to which available cues support either is variable and particularly so across VR scenarios. For example, in VR idiothetic cues supporting continuous updating are missing if self-motion is just simulated but available if real self-motion happens in parallel. Furthermore, VR environments can be sparse with only little spatial reference or they can provide rich spatial context encompassing boundaries and landmarks that support continuous and instantaneous updating. Spatial reference close to target locations is particularly valuable for instantaneous updating of egocentric target location memory.

### 2.2. Dynamic and static visual and non-visual cues

Visual and non-visual cues are contributing to spatial updating in the real world (Gallistel, 1990). As dynamic visual information, optic flow signals self-motion while self-motion takes place and supports continuous spatial updating. As static visual cues, boundaries and landmarks support instantaneous spatial updating. They afford encoding distances and directions to oneself, among them, and to other objects at any time (McNamara, 2013). Instantaneous spatial updating enables locating oneself instantly within an environment, for example, when waking up in a moving vehicle, when putting on a head-mounted display (HMD), or after teleportation in VR. Locations encoded with reference to static landmarks can also be instantaneously updated by updating or (re-)establishing the respective egocentric location representation (the box next to the fire hydrant or the buried coin next to the rock).

Continuous spatial updating during self-motion in the real world is usually supported not only by optic flow but also, in addition, by non-visual cues. These non-visual cues encompass vestibular information, proprioceptive information, and motor efference, and are referred to as body-based or idiothetic cues (Jeffery and O'Keefe, 1999; Chrastil and Warren, 2012). Idiothetic cues to self-motion allow for continuous spatial updating when optic flow is unavailable, for example, with closed eyes or in complete darkness (Loomis et al., 1993).

Continuously updating the ego-location and ego-orientation in navigation based on self-motion cues (including idiothetic cues and optic flow) is commonly called path integration, and instantaneously determining and updating ego-location and orientation with reference to static visual cues is named piloting. Research on self-localization and navigation in VR pertains to the updating of egocentric representations of locations. Generally, in sparse environments (or darkness or in a moving crowd), updating egocentric representations (self to environment and self to object) for reproducing object locations after self-motion is particularly important compared to the alternative of using encoded allocentric (object to environment) relations (cf. Cheng et al., 2007).

### 2.3. Passive and active translation and rotation in virtual reality

Translation denotes self-motion that changes the ego-location in an environment, and rotation denotes changing the heading direction even without changing location. When being translated or rotated passively in VR without actual body movement, optic flow is available but idiothetic cues to self-motion are missing. Rotation passively experienced in VR and thus without idiothetic cues results in marked performance impairments in tasks testing for the updating of spatial representations across self-motion compared with conditions, in which rotation is actively performed (Chance et al., 1998; Klatzky et al., 1998; Kearns et al., 2002; Wraga et al., 2004; Ruddle and Lessels, 2006; Cherep et al., 2020; Kelly et al., 2022; for an exception in a rich VR environment, see Riecke et al., 2007).

A common spatial updating task that involves rotation and translation is triangle completion, which requires navigating back to the starting location after traveling two outward legs of a triangle (or more than two legs, e.g., Kelly et al., 2008). For instance, Kearns et al. (2002) let participants who wore an HMD perform a triangle completion task in VR and compared joystick-controlled locomotion with real walking. They found superior performance for real walking. A second common task informing about the precision of spatial updating is pointing to a location after movement. Participants may be asked to point to or orient toward the starting location (e.g., Klatzky et al., 1998), which highlights the similarity of pointing with triangle completion (Wang, 2017). Instead of pointing to the starting location, pointing after movement can ask for pointing to locations of objects that are no longer visible at the time of testing (e.g., pointing after passive forward translation in Wolbers et al., 2008). For pointing to object locations after the movement that encompassed rotation and translation, the superiority of real walking compared to joystick-controlled locomotion in VR wearing an HMD has been demonstrated, for example, by Chance et al. (1998). If the task is not just spatial updating for a limited number of target locations and if the task requires the learning of an environment's layout for forming an accurate cognitive map and is tested, for example, by extended search tasks, in which revisits need to be avoided for efficiency (Ruddle and Lessels, 2006), or by collecting direction and distance estimates for multiple target locations after extended navigation in an environment (Ruddle et al., 2011), then real walking providing idiothetic cues about translation results in a clear advantage over conditions with passive translation. However, for spatial updating tasks not requiring learning a cognitive map, idiothetic cues to translation seem to be less important than idiothetic cues to rotation.

While being passively rotated impairs spatial updating performance reliably, mere passive translation is not consistently detrimental. Passive translation combined with real rotation can result in similar performance as real walking (Klatzky et al., 1998; Riecke et al., 2010; Barhorst-Cates et al., 2021) or at least in better performance than if both translation and rotation are joystick-controlled (Chance et al., 1998). Optic flow during translation is critical, however; teleportation impairs spatial updating consistently (Cherep et al., 2020; Barhorst-Cates et al., 2021; Kelly et al., 2022).

Passive translation in sparse environments may be easier to account for in updating than passive rotation (e.g., in reproducing the location of a target object) either because continuous online updating needs fewer sensory cues for translation than for rotation or because instantaneous offline updating is easier for translation than rotation (Hodgson and Waller, 2006). It may be easier in forward translation because the egocentric reference frame remains aligned with encoded object-to-object relations (or more generally, an allocentric reference frame). Offline updating of a target location after translation can then be performed by determining the current self-location within the allocentric frame and the line connecting the self with the target location. The orientation of this line is the direction for pointing to the target location. The distance for pointing can be determined by imagining a parallel to the forward axis through the target location in the allocentric frame and locating its intersection with the pointing direction. Such use of aligned egocentric and allocentric representations also explains that updating for merely imagined translation is much easier than for imagined rotation (Rieser, 1989; Presson and Montello, 1994).

But passive translation may also be easier to process than passive rotation just because continuous spatial updating for forward translation needs fewer sensory cues and can succeed with optic flow alone. To test whether optic flow in passive translation is sufficient for continuous spatial updating for forward translation is interesting for interpreting fMRI results obtained with participants seeing optic flow suggesting forward translation while lying supine in an MRI scanner and performing a spatial memory and pointing task similar to the one employed in the present experiments (Wolbers et al., 2008). If optic flow alone would be sufficient and idiothetic cues by real walking would not improve performance, then this would support the claim that this and similar fMRI studies inform about brain activation in spatial updating in real scenarios. Succeeding continuous spatial updating driven by optic flow during passive translation would directly provide the novel egocentric location of the target object. In any case, continuous spatial updating should reliably succeed if it is driven additionally by idiothetic cues from real walking during active translation. Thus, richer sensory cues could improve spatial updating across forward self-motion in VR.

In Experiment 1, we tested for an effect of pronounced optic flow during passive translation on pointing performance in reproducing object locations after self-motion, and in Experiment 2, we tested for an effect of real walking by comparing passive and active translations. To preview our results on these manipulations, we did not find reliable performance improvements from increased optic flow and real walking compared to passive translation. In addition, we studied the effects of landmarks and boundaries as static visual cues that provide a reference for locating oneself as well as for encoding object locations (and that—present in the field of view during self-motion—also increase optic flow).

### 2.4. Landmarks and boundaries

Landmarks and boundaries, for instance, static objects, corners, texture borders, and walls, provide spatial reference for self-localization and for encoding and reproducing target locations. Particularly in familiar environments, humans can determine their location and the egocentric relations to (earlier encoded) object locations by estimating distance and bearing to visible reference. Such instantaneous spatial updating is also employed to correct for otherwise cumulating errors in continuous spatial updating (Ekstrom et al., 2018).

Encoding and updating occur in parallel in allocentric and egocentric reference frames (Mou et al., 2004; Byrne et al., 2007). Boundaries and landmarks provide the spatial context for allocentric self-localization, updating, and episodic spatial memory as extensively documented by neuroscientific findings on place cells, grid cells, boundary vector cells, and head direction cells in animals and humans (Evans et al., 2016). More recently, neuroscientific evidence accumulated for egocentric self-localization and updating as well: Corresponding to recent findings in animal studies and predictions of neurocomputational models of human spatial cognition, single-cell recordings in humans have documented egocentric bearing cells prevalent in the parahippocampal cortex (Kunz et al., 2021). In the experiments, participants navigated with arrow keys within a circular boundary surrounded by landmarks in a virtual environment experienced on a laptop screen while performing a spatial memory task. The firing rates of egocentric bearing cells encoded the egocentric direction toward reference points in the present spatial context. For instance, the firing rate could be particularly high if the reference point was located to the left of the current heading direction. Some egocentric bearing cells did not just code for egocentric direction but also for egocentric distance and thus supported a vectorial egocentric representation of the local environment. Of particular interest for the present study, the tuning of egocentric bearing cells persisted during passive self-motion in the virtual environment, which presumably provides a neural mechanism for egocentric continuous spatial updating in the local spatial context of a visually rich environment even without idiothetic cues.

Landmarks and boundaries constitute the spatial context for encoding and updating in allocentric and egocentric reference frames. Compared to an open field, boundaries can alleviate the impairment by missing idiothetic cues when a triangle completion task is performed in VR with teleportation (Cherep et al., 2020). Objects as landmarks that were located at the boundary have been found to further improve performance in a triangle completion task (Kelly et al., 2022). Boundaries have been compared to landmarks in spatial learning with the presumption that they may be processed fundamentally differently in spatial memory (Doeller and Burgess, 2008); however, the currently available evidence rather supports the view that both function as environmental cues contributing to spatial context (Buckley et al., 2021).

Shapes of boundaries and configurations of landmarks can cause and influence biases in spatial memory and updating (Kelly et al., 2008, 2013; McNamara, 2013; Zhou and Mou, 2019). For instance, in a recent spatial memory experiment in VR, either a rectangular boundary consisting of three walls or three traffic cones as landmarks (located at the center points of the walls and forming a triangle) were provided as spatial context (Negen et al., 2020). Participants were teleported to a new and rotated viewpoint between encoding and retrieval. The raw average error did not differ between conditions, but the observed bias pattern in retrieved target locations differed between the wall (rectangle) and the cone (triangle) conditions. With walls, the target locations were retrieved with biases toward a point in front of the center of the front wall. With cones as landmarks, the target locations were retrieved with biases toward a point at the center of the triangle formed by the cones and slightly more distant from the front cone. In both conditions, the biases were stronger the more distant a target location was from the respective point. We studied such boundary and landmark effects on bias and precision in more detail.

In Experiment 1, we tested for effects of a (partial) boundary and landmarks at the boundary by presenting a lateral wall containing distinctive local features that could function as landmarks. In Experiment 2, we were interested in comparing boundaries with landmarks closer to target locations (inside the boundary) and in the combined effects of boundaries and such landmarks (cue combination). We presumed that landmarks closer to target locations could compensate for the lack of idiothetic cues across passive self-motion in VR even more effectively than boundaries. Furthermore, in Experiment 2, we varied boundary shapes (rectangle, trapezoid, and ellipse) comparing shapes with and without vertices that can function as landmarks and shapes with sides parallel to or oblique to the forward axis of translation. Thus, we varied spatial context in its richness which is presumably important for supporting continuous updating, and by the number and proximity of landmarks to targets in its usefulness for instantaneous updating.

## 3. Experiment 1—Lateral wall as reference and sleepers for optic flow

In Experiment 1, participants encoded two target locations in immersive VR, experienced passive forward translation, and reproduced one target location by pointing. We varied visual cues that potentially could support in updating. A stripe pattern resembling railway sleepers presented overhead only during translation was intended to increase optic flow. A lateral wall with distinctive local features was intended to provide spatial reference, particularly for encoding and reproducing target locations close to the lateral wall. We assessed updating performance by distance error, bias, and precision.

### 3.1. Materials and methods

#### 3.1.1. Participants

Thirty-two students of the Chemnitz University of Technology (15 men, 17 women) with a mean age of 25.36 years (*SD* = 4.84) participated in two sessions separated by 1–3 days (*M* = 1.41, *SD* = 0.70) that lasted ~90 min each. They fulfilled part of a curricular requirement or received monetary compensation (30 Euros). Two additional participants did not complete the experiment because of VR motion sickness. The data of four additional participants were excluded because of technical or experimenter errors that caused incomplete or unbalanced datasets. The data and analysis scripts are publicly available: https://osf.io/e39jf/.

#### 3.1.2. Design

All experimental factors varied within subjects. Trials with translation (Translation) and trials without translation (No Translation) were intermixed. In Translation trials, either overhead sleepers were shown during translation or not (Sleepers and No Sleepers). The environment either was sparse (No Wall) or contained a lateral wall that was presented either left or right. To-be-reproduced target locations were also either left or right and consequently were close (Same Side) or distant (Opposite) to a lateral wall. For Translation trials, this resulted in a 2 × 3 factorial within-subjects design including the factors Sleepers (Sleepers and No Sleepers) and Wall (Same Side, Opposite, and No Wall). For No Translation trials, the design was one-factorial within-subjects with the three-level factor Wall (Same Side, Opposite, and No Wall).

Performance in reproducing target locations was quantified by the mean absolute Euclidean distance between the indicated and the actual target location on the floor plane (absolute distance error), by the mean signed distances on the x-axis and the forward y-axis (response bias), and by the standard deviations of the distances on the x-axis and the forward y-axis (lower values indicating higher precision).

#### 3.1.3. Apparatus and stimuli

The virtual environment was presented stereoscopically in an Oculus Quest head-mounted display (HMD) with a resolution of 1,440 × 1,600 pixels per eye (71 Hz frame rate, 94° × 94° field of view). Tracking of the HMD and a hand-held controller used a combination of inside-out tracking by four cameras and sensors including a gyroscope and acceleration sensors. Unity and the Oculus Link were used to render images on a Notebook with the Intel i7 processor and an NVIDIA Quadro RTX 3000 graphics card.

**Figure 1:**
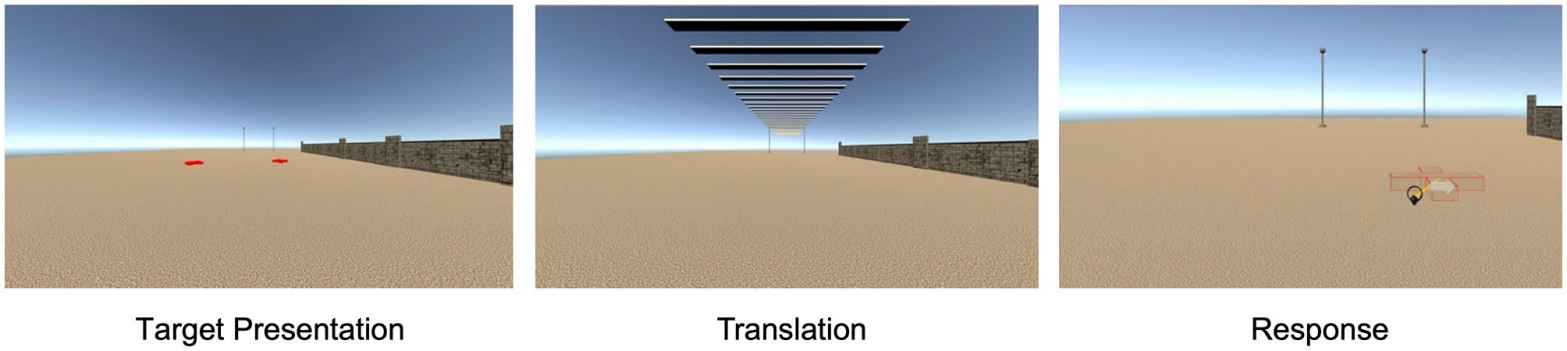
A virtual environment in Experiment 1 with a lateral wall on the right and sleepers during translation.

The virtual environment was an open field scene with a light ochre sand-textured floor plane extending to the horizon under a cloudless atmospheric blue sky (Figure 1). The participant's perspective was set at a height of 180 cm. The participant at coordinates (0, 0) on the left-right and front-back axes, respectively, was oriented toward a pair of 7 m high poles visible ahead at (−5 m, 40 m) and (5 m, 40 m). In the No Wall condition, these were the only reference objects in the environment. In the trials with a lateral wall (Same Side and Opposite), a 2-m high brick wall extended parallel to the forward axis 11 m to the left or the right of the participant almost up to the poles ending at (−11 m, 31 m) or (11 m, 31 m), respectively. In total, 12 different wall shapes (mirrored for left and right versions) were constructed by attaching flat columns of varying height at intervals of around 5 m at the side facing the participant creating protrusions and crenels. In the Sleepers condition, a cross-striped rectangular surface that extended as a band overhead the participant to the poles was presented during forward translation to strengthen optic flow. The sleepers band was 4.6 m wide at a height of 4.2 m with stripes of 0.2 m alternating in light and dark gray. In trials with translation, the passive forward translation was 7, 8, or 9 m.

**Figure 2:**
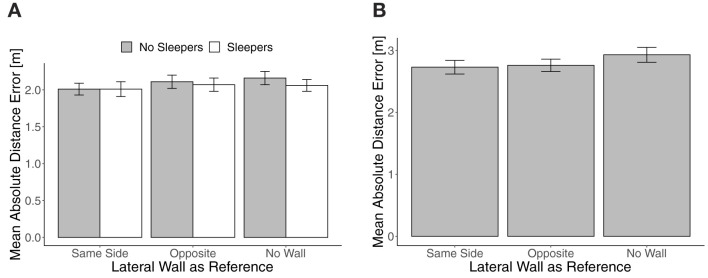
Mean absolute distance error in Experiment 1 in trials with translation **(A)** and trials without translation **(B)**. Error bars show standard errors of the mean.

Objects at target locations were red cross shapes (1.5 × 1.5 m) with bars of breadth 0.4 m and height 0.25 m. In each trial, two such target objects were presented, one to the left and one to the right of the central forward axis. Thus, in trials with a lateral wall, one was close to a wall (Same Side) that provided spatial reference for encoding and reproducing the location and one was distant to a wall (Opposite). Target locations were drawn from bivariate Gaussian distributions (*SD*s 1 m) centered at four points on the left (L1, L2, L3, and L4) and four corresponding points at the right (R1, R2, R3, and R4) shown in Figure 2 and Table 1. All pairs were non-symmetric (e.g., L1 and R2, but not L1 and R1).

**Table 1:** Target locations in Experiment 1.

**Target location**	**Left**	**(x, y)**	**Right**	**(x, y)**
1	L1	(−6, 17)	R1	(6, 17)
2	L2	(−3, 15)	R2	(3, 15)
3	L3	(−3, 13)	R3	(3, 13)
4	L4	(−6, 11)	R4	(6, 11)

The participants responded with the tracked controller. They directed an orange laser beam and a red wireframe model of a target object that was presented where the laser beam intersected with the floor plane. At the laser beam 1.3 m from the controller, an arrow pointing to the right or the left indicated which target location should be marked by placing the wireframe model.

#### 3.1.4. Procedure

Upon arrival for the first session, the participant was informed about the procedure of both sessions and signed informed consent. The experimenter provided instructions on how to put on the head-mounted display (spatial distancing during the SARS-CoV-2 pandemic). The participant stood upright while wearing the HMD and held the controller in the right hand.

The task was introduced with 12 training trials with feedback followed by 12 training trials without feedback. Training trials were balanced with regard to conditions and stimuli. The participant started a trial by pressing the trigger button on the controller (operated by the index finger). After a black blank screen was shown for 200 ms, an auditory signal was played and two target objects were presented in the virtual environment for 5 s, which then sank into the ground for 2 s. In trials without translation, the scene without the targets remained static for 7 s. In trials with translation, the scene without the targets remained static for 7 s followed by the passive forward translation lasting 3,500, 4,000, or 4,500 ms for 7, 8, and 9 m, respectively. Subsequently, a second auditory signal marked the start of the response interval during which the laser beam with the red wireframe cross and the arrow identifying the target location to be indicated were shown until the participant responded by pressing the controller button. The laser beam and arrow disappeared and the placed wireframe remained visible for 1,500 ms. Then, the viewpoint was set back to the starting location, and a message window prompting to start the next trial was shown hovering in front of the poles with the wireframe still visible on the ground. In training trials with feedback, the target objects were shown after the response together with the placed wireframe. In trials with sleepers, the stripe pattern was shown during the translation interval.

Separated into two sessions, participants responded once for each of the 8 target locations in each possible combination of condition levels. There were 24 combinations when discerning lateral wall placement left and right which determines whether a target location is close or distant to a wall: 6 (NoWall, Same Side, Opposite, NoWall/Sleepers, Same Side/Sleepers, and Opposite/Sleepers) × 4 (translation 0, 7, 8, 9 m). Please note that at translation 0, sleepers were never shown. This resulted in 192 (8 × 24) experimental trials in total, which were presented in eight blocks (four blocks in the first and four blocks in the second session) and in pseudorandomized order, ensuring that condition levels were distributed evenly across the trial sequence, condition levels did not repeat more than once in subsequent trials, and that no more than two subsequent to-be-reproduced target locations were on the same side. Between blocks, participants took the HMD off for a break.

When returning to the lab for the second session, the participant first took computerized mental rotation and spatial orientation tests on a laptop (not further reported). Then, the participant was offered to repeat training trials before working through four blocks of experimental trials following the same procedure as in the first session.

### 3.2. Results

Trials were retained for analysis if responses were to the correct side, the pointing error was not higher than 6 m of absolute Euclidean distance from the target location, and the response time was <8 s (98% of all trials). In repeated-measures ANOVAs, *F*-values have been Greenhouse–Geisser corrected where necessary.

#### 3.2.1. Absolute distance error

Mean absolute distance error in trials with translation did not differ between trials with targets to the left and the right (no reliable main effect of target side, *F*_(1, 31)_ = 2.30, *p* = 0.14, and no reliable two- or three-way interaction of the sleepers and the lateral wall manipulations with target side, all *F*s <1.47, all *p*s > 0.24). As shown on the left in Figure 2, mean absolute distance error in translation trials was smallest with a lateral wall at the Same Side. It slightly increased from Same Side to No Wall when No Sleepers were shown and was slightly decreased with Sleepers in Opposite and No Wall conditions. In a repeated-measures ANOVA, neither the main effects of Sleepers, *F*_(1, 31)_ = 1.28, *p* = 0.27, $\eta_{\text{p}}^{2}$ = 0.04, and the lateral wall manipulation, *F*_(2, 62)_ = 1.07, *p* = 0.33, $\eta_{\text{p}}^{2}$ = 0.03, nor the interaction effect, *F*_(2, 62)_ = 0.83, *p* = 0.44, $\eta_{\text{p}}^{2}$ = 0.03, were reliable. In the No Wall condition, the Sleepers effect was largest, but unreliable as well, paired *t*-test *t*_(31)_ = 1.48, *p* = 0.15, *d* = 0.20.

The mean absolute distance error in trials without translation is shown on the right in Figure 2 and also increased slightly from Same Side to No Wall. In a repeated-measures ANOVA, the effect of the lateral wall manipulation was unreliable, *F*_(2, 62)_ = 2.87, *p* = 0.07, $\eta_{\text{p}}^{2}$ = 0.09. The effect of a lateral wall on the Same Side vs. No Wall was *d* = 0.27, paired *t*-test *t*_(31)_ = 1.78, *p* = 0.09 to quantify the largest difference.

#### 3.2.2. Bias and precision

**Figure 3:**
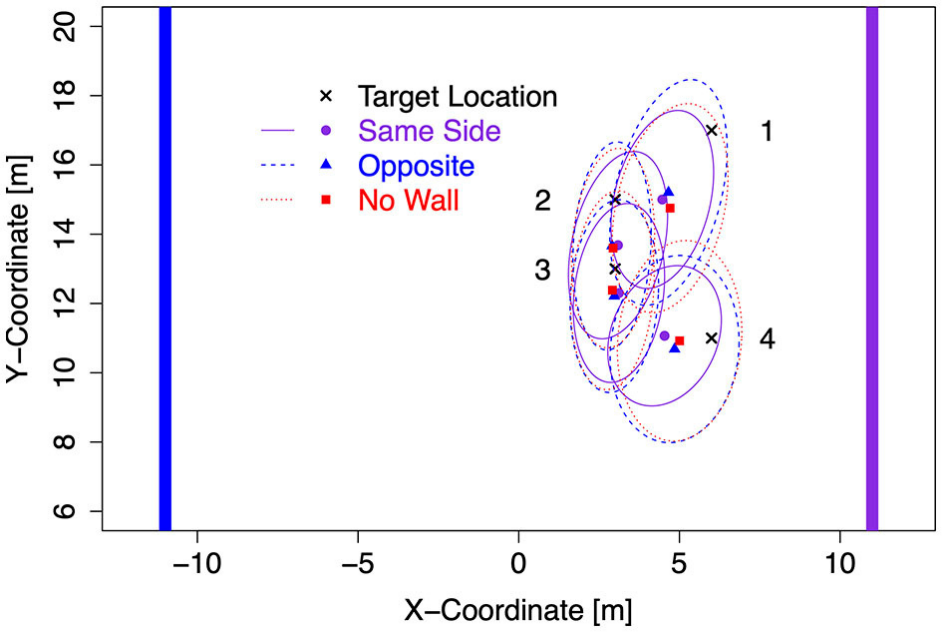
Mean response coordinates with ellipses capturing about 80% of responses in Experiment 1.

For the subsequent analysis of response bias (signed distance error) and response precision in translation trials, first, data were collapsed across translation trials with and without sleepers. Second, data for left target locations were flipped on data for right target locations (after recentering both to correct for the bivariate Gaussian variation). Mean response coordinates are shown in Figure 3 for each of the four target locations separately for Same Side, Opposite, and No Wall conditions with ellipses capturing about 80% of the responses (a figure showing left and right data separately is provided in the Supplementary material, and a figure showing the data for No Translation trials is provided there as well). As visible, bias in pointing responses revealed the more underestimation of distances the more distant target locations were. Furthermore, an inward bias was apparent for the more lateral target locations 1 (L1/R1) and 4 (L4/R4). Precision was higher for these lateral target locations with a lateral wall on the Same Side as indicated by smaller ellipses.

To quantify bias on the x- and y-axes as well as precision at each level of the lateral wall manipulation, Bayesian estimation was applied for modeling pointing responses separately for each target location as bivariate normally distributed and grouped by a nominal predictor. For x- and y-coordinates, intercepts and group deflections were estimated together with group-specific covariance matrices, from which the rotation angle θ of data ellipses could be computed. Intercepts are denoted β_x0_ and β_y0_, respectively. Group deflections are denoted β_x_[i] and β_y_[i] with i varying from 1 to 3 for the levels of the lateral wall manipulation. Both β_x_[i] and β_y_[i] sum to zero.

Bayesian estimation was implemented in R and JAGS (Plummer, 2003) by combining and adapting a script for a single normally distributed variable with one nominal predictor provided in Kruschke (2015) and a script for estimating parameters of a bivariate normal distribution provided by Bååth (2013), following their recommendations for non-committal priors. The R script and data files for replicating the analysis are provided in the OSF.

**Figure 4:**
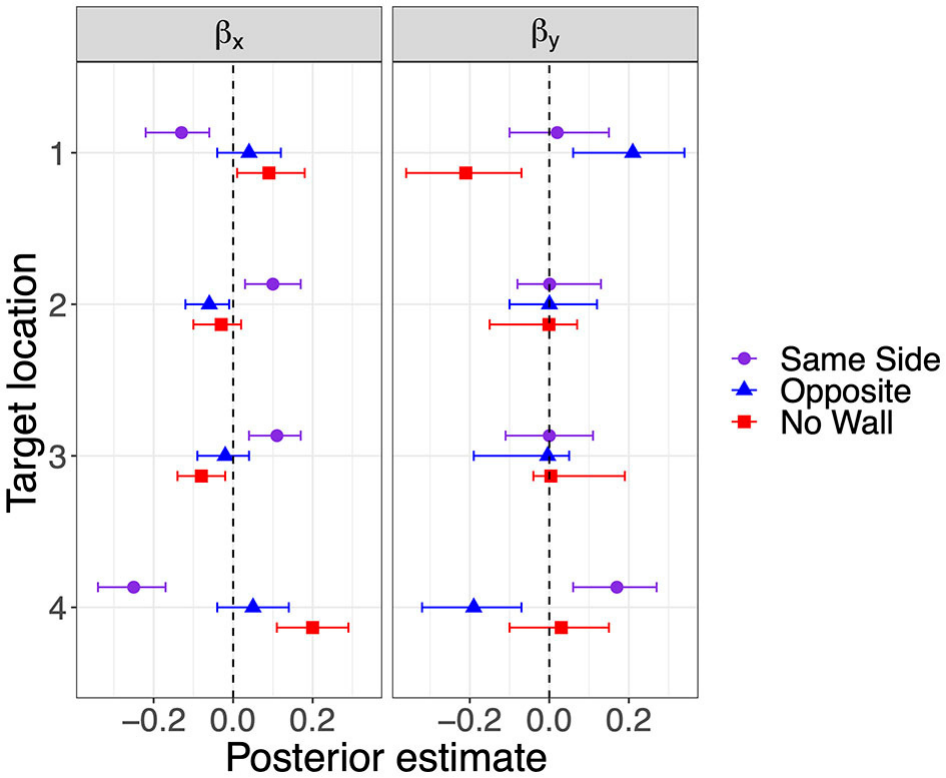
Modes and 95% HDI boundaries for deflection parameter estimates of bias on the x-axis and the forward y-axis in Experiment 1.

The modes and the lower and upper boundaries of 95% highest-density intervals of posterior distributions of parameter estimates are shown in Table 2 for the intercepts β_x0_ and β_y0_ and rotation angles. Figure 4 shows modes and 95% HDI boundaries for the group deflections β_x_[i] and β_y_[i] and standard deviations σ_x_[i] and σ_y_[i]. Underestimation of distances on the forward axis increasing with the distance of target locations is confirmed in increasingly negative values of β_y0_ from target location 4 to target location 1 with non-overlapping HDIs (Table 2). The bias in the x-direction reflected in β_x0_ was inward (negative signed error) for the more lateral target locations 1 and 4 and close to zero for target locations 2 and 3. A lateral wall on the same side pushed responses for the lateral target locations 1 and 4 even slightly more inward as shown by negative β_x_ for Same Side in Figure 4. In contrast, responses for target locations 2 and 3 seemed to be pulled slightly outward as indicated by positive β_x_ for Same Side.

**Table 2:** Parameter estimates of intercepts for bias on the x-axis and on the forward y-axis and rotation angles of response point clouds in Experiment 1.

	**PSRF**	**ESS**	**Mode**	**HDI low**	**HDI high**
**Target location 1 (L1/R1)**					
β_x0_ intercept	1.00	124,216	−1.39	−1.45	−1.33
β_y0_ intercept	1.00	137,363	−2.01	−2.11	−1.92
θ Same Side	1.00	84,336	15.72	10.76	20.54
θ Opposite	1.00	80,750	15.99	12.26	19.57
θ No Wall	1.00	80,920	14.67	10.17	19.20
**Target location 2 (L2/R2)**					
β_x0_ intercept	1.00	132,341	−0.02	−0.07	0.02
β_y0_ intercept	1.00	138,598	−1.36	−1.45	−1.26
θ Same Side	1.00	79,770	14.39	10.15	18.27
θ Opposite	1.00	91,731	3.63	0.82	6.25
θ No Wall	1.00	91,834	4.48	1.18	7.67
**Target location 3 (L3/R3)**					
β_x0_ intercept	1.00	143,383	−0.00002	−0.04	0.04
β_y0_ intercept	1.00	146,976	−0.70	−0.79	−0.61
θ Same Side	1.00	88,253	7.77	3.63	12.19
θ Opposite	1.00	90,820	4.10	0.31	8.03
θ No Wall	1.00	88,402	4.42	1.34	7.52
**Target location 4 (L4/R4)**					
β_x0_ intercept	1.00	146,452	−1.20	−1.27	−1.14
β_y0_ intercept	1.00	125,513	−0.11	−0.19	−0.02
θ Same Side	1.00	89,702	29.25	18.05	40.29
θ Opposite	1.00	88,757	7.22	−1.69	16.79
θ No Wall	1.00	92,934	6.33	−0.51	13.35

PSRF, Potential Scale Reduction Factor; ESS, Effective Sample Size; HDI low and HDI high, Lower and upper boundaries of 95% highest density intervals.

**Figure 5:**
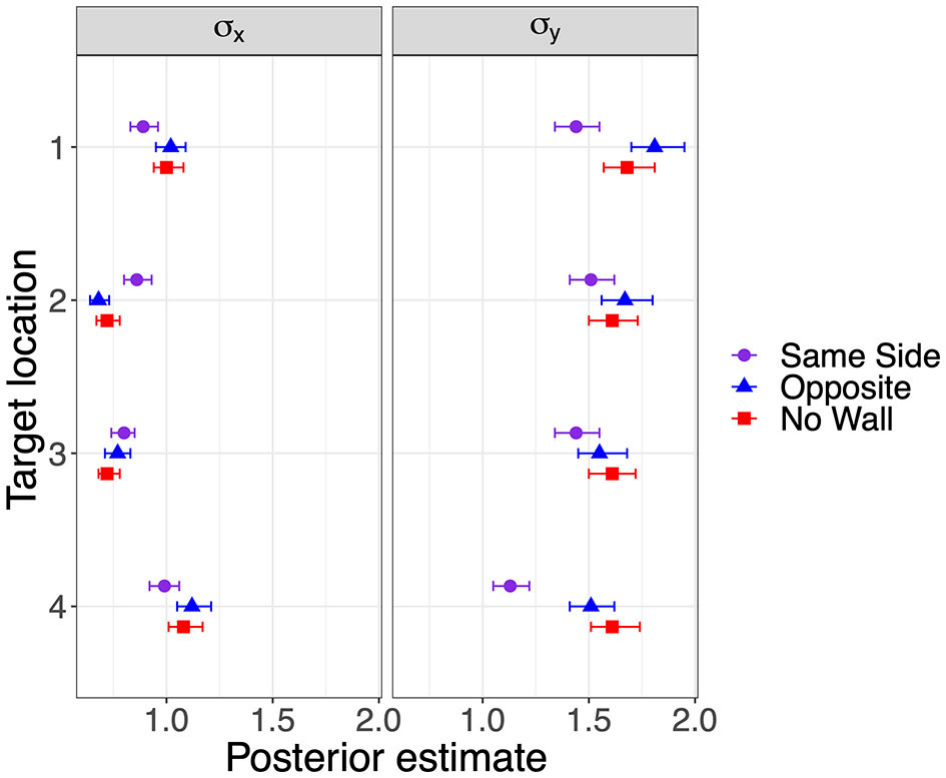
Modes and 95% HDI boundaries for parameter estimates of standard deviation on the x-axis and on the forward y-axis in Experiment 1.

Response precision has been estimated by the standard deviations shown in Figure 5. Lower standard deviations on the forward axis σ_y_ corresponding to higher precision were observed for the lateral target locations 1 and 4 when a lateral wall was on the Same Side, most clearly for the closer target location 4. Standard deviations in the x-direction did not show consistent effects of the lateral wall manipulation but were also slightly decreased for the lateral target locations 1 and 4 in the Same Side condition.

The estimates for the rotation angle of the response point clouds (Table 2) turned out higher with a lateral wall on the Same Side for all but the most distant target location 1 (consistent with a stronger inward bias for less distant locations). For target locations 2 and 4, the Same Side HDIs for θ are clearly above and non-overlapping with the HDIs for Opposite and No Wall, which are highly overlapping for all target locations.

### 3.3. Discussion

The overall analysis of absolute distance error did not reveal reliable effects of the sleeper (optic flow) manipulation and the spatial reference provided by a lateral wall with distinctive features as landmarks. However, slight trends were consistent with the presumed updating-supporting effects of increased optic flow and spatial reference. Effects of spatial reference showed up for target locations close to the lateral wall. Bayesian estimation of signed error on the x- and y- (forward) axes allowed us to quantify bias and precision at different levels of the spatial reference manipulation separately for the four target locations (not assuming variance homogeneity as in standard ANOVAs). The results confirmed the well-known phenomenon of distance underestimation in VR (Renner et al., 2013; Vienne et al., 2020), which increased with target distance (evident in y- and x-intercepts). In addition, results showed clearly that the effects of the spatial reference manipulation were restricted to the two target locations close to the lateral wall: the target location 1 furthest from the participant and the target location 4 closest to the participant. The lateral wall increased the precision of the pointing responses, particularly by reducing variance on the y-axis and pushing pointing responses slightly inward only for the two target locations close to the lateral wall. The inward bias is consistent with previous results of Negen et al. (2020), who also found that target locations were reproduced with a bias inward away from close boundaries.

The optic flow manipulation may have turned out more influential if all other visual cues potentially processed for updating would have been minimized. This is suggested by the small and unreliable Sleepers effect without any wall. The lateral wall if present did not only provide static reference but also increased optic flow and thus may have diminished the Sleepers effect. We did not study optic flow without persisting visual cues (e.g., by flaring random dots or fiery textures) because our interest in effective support for spatial updating in VR is motivated partly by such support's relevance in applied contexts such as teleoperation of vehicles and mobile robots and these very sparse environments are uncommon. Environments typically contain boundaries and landmarks or at least, they could be added in VR or added by augmented reality techniques to immersive teleoperation interfaces (Suzuki et al., 2022). Hence, we strengthened support and investigated the effects of boundaries and landmarks separately and in combination in Experiment 2 along with idiothetic cues from real walking. Landmarks were placed inside the boundary close to target locations.

## 4. Experiment 2—Landmarks and boundaries with translation and walking

Again, participants encoded two target locations, one of which had to be reproduced after forward self-motion. We varied whether only a boundary was available as spatial context, or instead five objects as close landmarks, or both a boundary and the five landmarks within the boundary. We expected that support in reproducing target locations and effects showing up in bias and precision should be stronger for target locations close to the boundary and/or landmarks as indicated in Experiment 1 and suggested by multiple previous studies (McNamara, 2013). When both boundary and landmarks are present, cue combination may produce particularly strong support as well as bias and precision effects (Newman and McNamara, 2022). In previous studies (e.g., Kelly et al., 2008), boundary shapes have been selected to vary the presence and alignment of main axes and the presence of vertices that could function as landmarks similar to objects (among other factors, e.g., rotational symmetry, which is relevant if self-motion encompasses rotation). The participants experienced one of three boundary shapes: a rectangle with two lateral walls aligned with forward translation, a trapezoid with lateral walls oblique to forward translation, and an ellipse with a longer axis aligned with forward translation. All three shapes had a main axis aligned with forward translation, but only the rectangle and the trapezoid contained vertices as potential landmarks. Target locations were placed in between the boundary and landmarks (thus being enclosed if both were present), and one was placed between landmarks to which it was closer than to the boundary. Enclosing could amplify the benefit of cue combination.

In addition to varying available spatial context and the placement of target locations, we let participants experience real walking instead of passive forward translation in half of the trials. Idiothetic cues from real walking were effective support for spatial updating in previous studies if self-motion included rotation (Klatzky et al., 1998; Kearns et al., 2002; Wraga et al., 2004; Ruddle and Lessels, 2006) and in a subset of studies also for forward translation (Chance et al., 1998; see Ruddle and Lessels, 2006 and Ruddle et al., 2011 for the unequivocal benefit of real walking for establishing a cognitive map). To test the potential supporting effects of real walking on forward updating of location memory in the context of boundaries and/or landmarks, we compared passive translation with real walking in Experiment 2.

### 4.1. Materials and methods

#### 4.1.1. Participants

Thirty-six students of Chemnitz University of Technology (16 men, 20 women) with a mean age of 25.94 years (*SD* = 4.87) participated in a single session that lasted around 180 min in partial fulfillment of a curricular requirement or for monetary compensation (10 EUR/h). Nine additional participants were replaced: One participant did not complete the study due to technical failure, two participants deviated often from the correct path in walking trials, and data of six participants had to be excluded due to technical errors in presenting the pointing task.

#### 4.1.2. Design

The experiment constituted a 3 × 3 × 2 mixed factorial design including boundary shape (rectangle, trapezoid, and ellipse) as a between-subjects factor, and environment (landmarks, boundary, and boundary and landmarks) and translation type (passive Translation and real Walking) as within-subjects factors.

#### 4.1.3. Apparatus and stimuli

**Figure 6:**
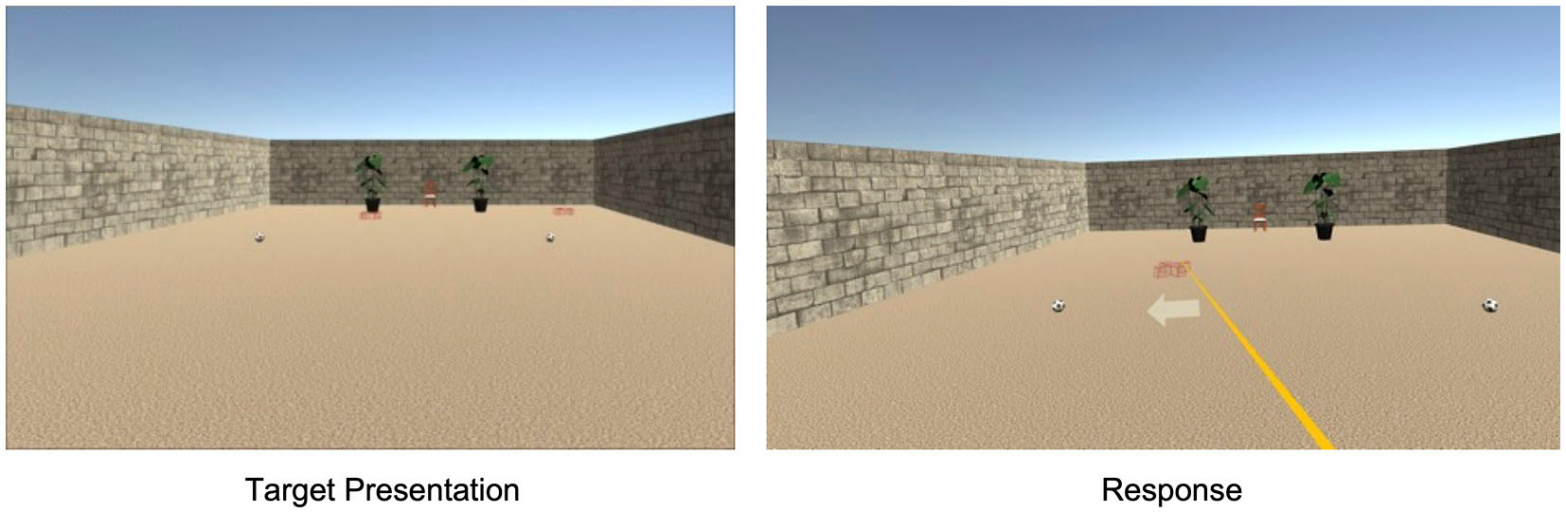
A virtual environment in Experiment 2 with landmarks and a rectangle boundary.

**Figure 7:**
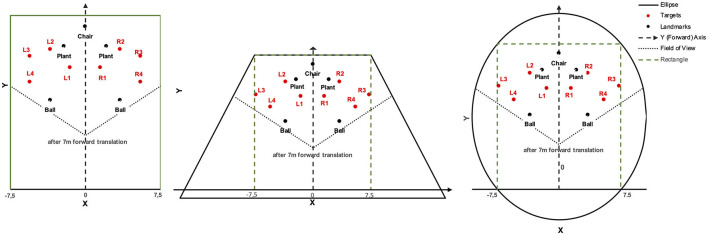
Boundary shapes with landmarks and target locations in Experiment 2.

As in Experiment 1, the virtual environment was a light-ochre floor plane under a cloudless blue sky (Figure 6). When only landmarks were presented, the floor plane extended to the horizon. Landmarks were a pair of soccer balls (20 cm high) spaced 7 m apart, a pair of plants (80 cm) spaced 4.2 m apart, and a chair (80 cm) tiered in front of the participant. Measured from the starting location of the participant at coordinates (0, 0) on the left-right and front-back axes, respectively, the chair was 21 m ahead. Boundaries were walls of 3 m height and enclosed the starting location and all landmark locations. The boundary shape was either a 15 m by 24 m rectangle, an isosceles trapezoid whose width extended from 15 to 34.35 m over a height of 25 m, or a 21.22 m by 31.80 m ellipse. The starting location in the rectangle was 1.5 m apart from the wall opposite the chair location. The trapezoid and the ellipse were constructed such that the part of the rectangle ahead of the starting location fitted inside as shown in Figure 7.

Target coordinates were drawn from bivariate Gaussian distributions (*SD* = 1 m) centered at four locations to the left and four mirrored locations to the right of the forward axis per boundary shape (Table 3). The target locations were chosen to be located either near at a distance of 9 m (locations 1 and 4) or far at 12.5 m (locations 2 and 3) from the participant's location at (0, 7) after 7 m forward translation. Furthermore, distances to landmarks and boundaries were considered. Target location 2 was 1.5 m away from the plant landmark and thus the closest to a landmark. Target location 3 was 1.5 m away from the boundary (with different coordinates for the rectangle than for the trapezoid and ellipse). Target location 4 was close to the boundary (1.5 m) only for the rectangle. All target locations were enclosed between landmarks (1) or between boundary and landmarks (2, 3, and 4). As in Experiment 1, two red cross shapes were presented as left and right target objects in each trial with non-symmetric target locations. The starting location was marked by a small red X-mark at the center of a white circle with a diameter of 3 m.

**Table 3:** Target locations in Experiment 2.

**Target location**	**Left**	**(x, y)**	**Right**	**(x, y)**
1	L1	(−1.5, 15.67)	R1	(1.5, 15.67)
2	L2	(−3.5, 18.01)	R2	(3.5, 18.01)
3 Rectangle	L3	(−5.5, 17.16)	R3	(5.5, 17.16)
3 Trapezoid, Ellipse	L3	(−7.3, 15.96)	R3	(7.3, 15.96)
4	L4	(−5.5, 13.87)	R4	(5.5, 13.87)

The translation was either passive translation or real walking for 5, 6, or 7 m. Participants again responded with the controller by placing a wireframe model showing where a laser beam intersected with the floor plane. An arrow on the laser beam indicated the side, for which the target object location was to be reproduced.

#### 4.1.4. Procedure

After being informed about the procedure of the experiment and the tasks to be performed, the participant signed informed consent and was instructed by the experimenter how to put on and adjust the head-mounted display and how to operate the controller. Then, the participant worked through 18 training trials experiencing three trials each for passive translation and then real walking first within the respective boundary, second with landmarks only, and then for boundary and landmarks combined. Different target locations than in experimental trials were used and feedback was provided as in the first half of training trials in Experiment 1. No feedback was provided in experimental trials.

The participant stood upright at the starting location and initiated a trial with the trigger button on the controller. A left and a right target object were presented and sank into the ground. Passive translation trials proceeded as in Experiment 1 with a translation interval of 2.8–3.1 s. After the response, the participant was passively turned and translated back to the starting location. In real walking trials, the onset of a melody right after the target objects disappeared prompted the participant to start and keep walking forward until a gong sound stopped walking. When lateral deviation exceeded 0.3 m, the melody was replaced by noise until walking was back on track. At the onset of the gong sound that stopped walking, the response interval started. After the response, a sign popped up for 2 s instructing the participant to “Please, turn around and go back.” When the participant reached the starting location in the white circle, a second sign popped up for 2 s instructing to “Please, turn around and proceed.”

Participants worked through six blocks of trials. Each block consisted of 24 trials for a particular combination of environment and translation type (e.g., landmarks and translation) and included one trial for each target location to-be-reproduced left and right with each translation distance (4 × 2 × 3). This resulted in a total of 144 experimental trials. The sequence of blocks was balanced according to a Latin square. The order of trials within a block was pseudorandomized ensuring that target locations were distributed evenly across the trial sequence, translation distances did not repeat more than once in subsequent trials, and no more than two subsequent to-be-reproduced target locations were on the same side. Participants took a brief break after each block.

### 4.2. Results

Again, trials were retained for analysis if responses were to the correct side, the pointing error was not higher than 6 m of absolute Euclidean distance from the target location, and the response time was <8 s (98% of all trials). In repeated-measures ANOVAs, *F*-values have been Greenhouse–Geisser corrected where necessary.

#### 4.2.1. Absolute distance error

**Figure 8:**
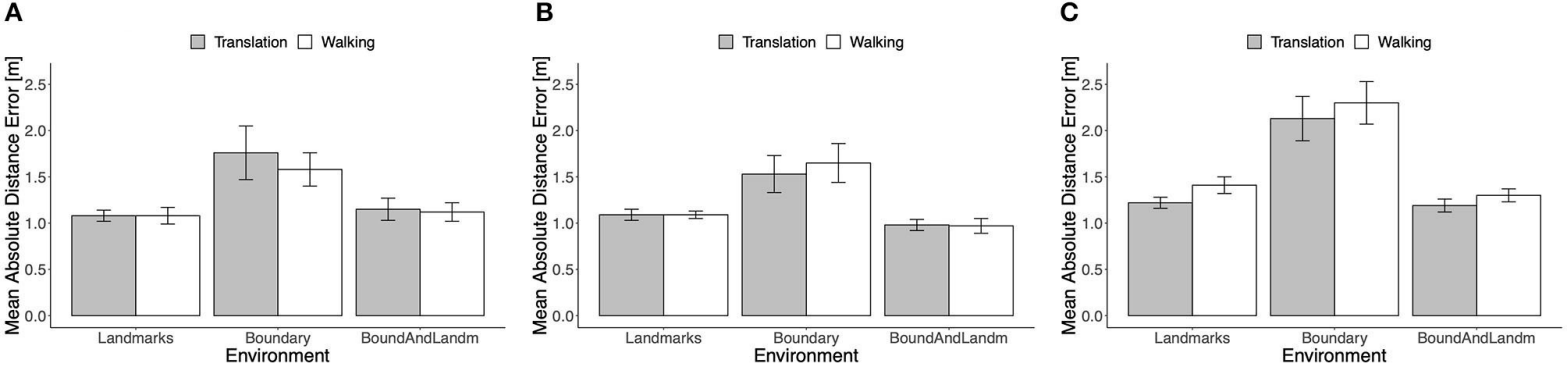
Mean absolute distance error in Experiment 2, **(A)** rectangle, **(B)** trapezoid, and **(C)** ellipse. Error bars show standard errors of the mean.

The mean absolute distance error is shown in Figure 8 separately for the three boundary groups. The type of self-motion had no consistent effect. In particular, the error in real walking trials was not consistently lower than in passive translation trials. In the Ellipse group, even the opposite trend was apparent across environmental conditions. With both types of self-motion and in all three groups, the mean error was higher in the Boundary condition than in the landmarks and boundary-and-landmarks conditions. Note, that in comparing performance across the groups, it has to be kept in mind that target location 3 was located more inward in the Rectangle than in the Trapezoid and Ellipse groups.

Repeated-measures ANOVAs including the factors type of self-motion and environment were computed separately for the three boundary groups. In the Rectangle group, there was no reliable main effect of self-motion type, *F*_(1, 11)_ = 1.20, *p* = 0.30, $\eta_{\text{p}}^{2}$ = 0.10, the main effect of environment was confirmed, *F*_(2, 22)_ = 8.85, *p* = 0.009, $\eta_{\text{p}}^{2}$ = 0.45, and there was no reliable interaction, *F*_(2, 22)_ = 0.76, *p* = 0.43, $\eta_{\text{p}}^{2}$ = 0.06. In the Trapezoid group, the pattern of effects was similar: no reliable main effect of self-motion type, *F*_(1, 11)_ = 0.59, *p* = 0.46, $\eta_{\text{p}}^{2}$ = 0.05, a clear main effect of environment, *F*_(2, 22)_ = 9.69, *p* = 0.007, $\eta_{\text{p}}^{2}$ = 0.47, and no reliable interaction, *F*_(2, 22)_ = 0.54, *p* = 0.52, $\eta_{\text{p}}^{2}$ = 0.05. In the Ellipse group, the unexpected advantage of translation over walking was confirmed by a main effect of self-motion type, *F*_(1, 11)_ = 8.55, *p* = 0.014, $\eta_{\text{p}}^{2}$ = 0.44. The main effect of environment was again reliable, *F*_(2, 22)_ = 17.55, *p* = 0.001, $\eta_{\text{p}}^{2}$ = 0.62, and again there was no indication of an interaction, *F*_(2, 22)_ = 0.23, *p* = 0.68, $\eta_{\text{p}}^{2}$ = 0.02.

In all three boundary groups, the mean absolute distance error was higher in the Boundary condition than in the Landmarks and Boundary-and-Landmarks conditions with *d* = 0.88, 0.84, and 1.43, and *d* = 0.61, 1.06, and 1.65 for the Rectangle, Trapezoid, and Ellipse groups, respectively. In the Rectangle group, distance error was similar in the Boundary-and-Landmarks and the Landmarks conditions, paired *t*-test *t*_(11)_ = 0.67, *p* = 0.52, *d* = 0.17, whereas in the Trapezoid and Ellipse groups, the error was slightly lower in the Boundary-and-Landmarks condition with *t*_(11)_ = −2.06, *p* = 0.06, *d* = −0.65, and *t*_(11)_ = −1.72, *p* = 0.11, *d* = −0.30, respectively.

#### 4.2.2. Bias and precision

**Figure 9:**
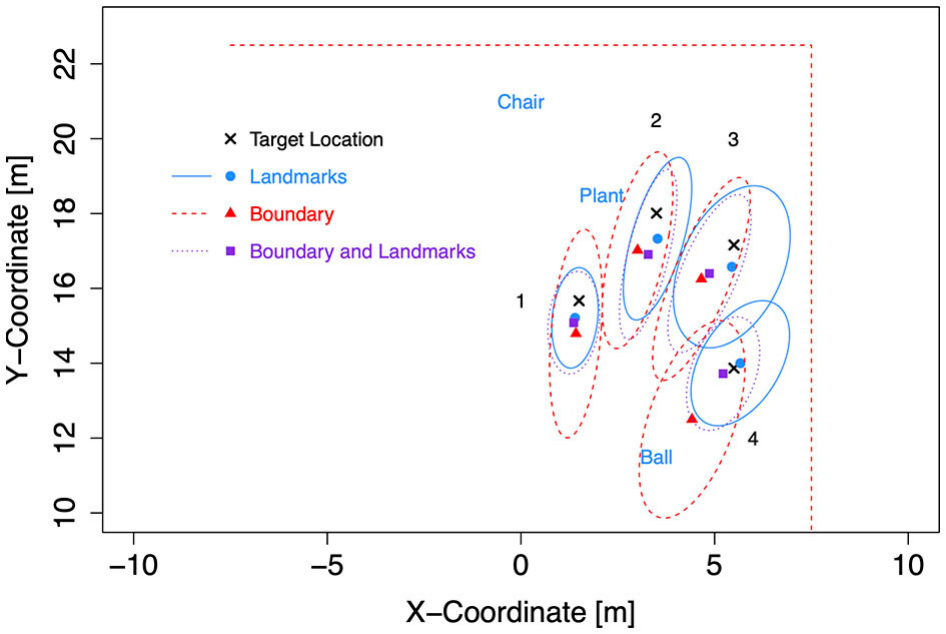
Mean response coordinates with ellipses capturing about 80% of responses for the Rectangle group in Experiment 2.

**Figure 10:**
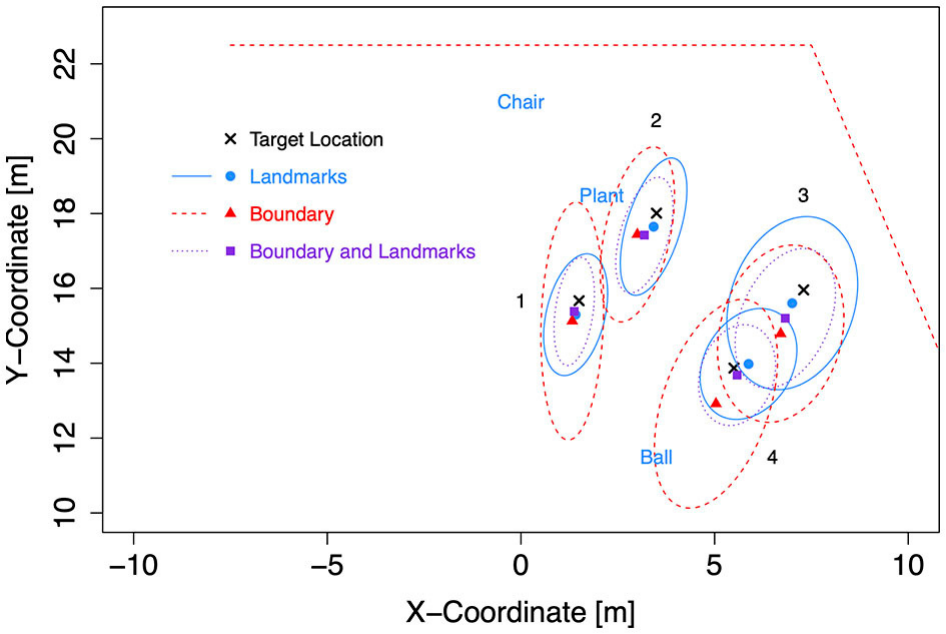
Mean response coordinates with ellipses capturing about 80% of responses for the Trapezoid group in Experiment 2.

**Figure 11:**
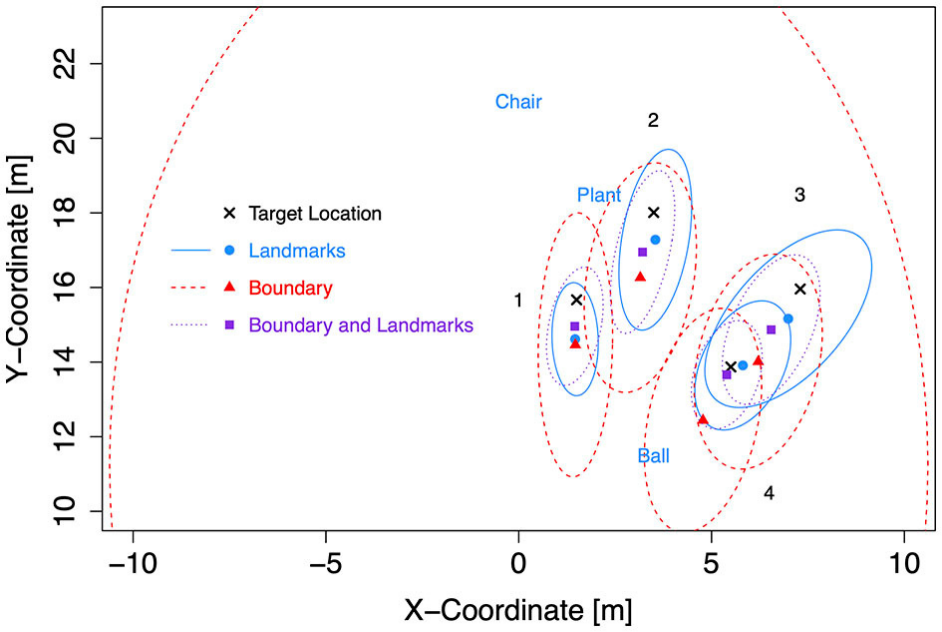
Mean response coordinates with ellipses capturing about 80% of responses for the Ellipse group in Experiment 2.

For the subsequent analyses of bias and precision, data for both self-motion types have been combined, because the effects of the self-motion type were inconsistent and small compared to the consistent and strong environment effect. As for the Bayesian analyses of Experiment 1, data for left target locations were flipped on data for right target locations (after recentering both to correct for the bivariate Gaussian variation). Mean response coordinates are shown in Figure 9 (Rectangle), Figure 10 (Trapezoid), and Figure 11 (Ellipse) for each of the four target locations separately for Landmarks, Boundary, and Boundary-and-Landmarks with ellipses capturing about 80% of the responses (figures showing left and right data separately are provided in the Supplementary material).

General patterns visible in the figures are, first, responses are pushed away from close boundaries, second, the presence of landmarks (in the chosen placement of targets, landmarks, and boundaries) reduced distance error and increased precision more than the presence of boundaries, and third, typically, the presence of both boundary and landmarks resulted in the highest precision. For target location 1, which was enclosed between landmarks and most distant from the boundary, the presence of a boundary in addition to landmarks did hardly increase precision further.

**Figure 12:**
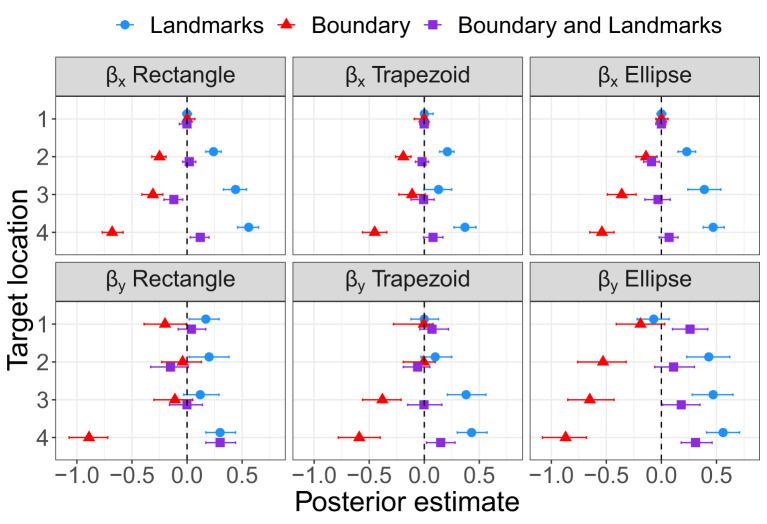
Modes and 95% HDI boundaries for deflection parameter estimates of bias on the x-axis and the forward y-axis in Experiment 2.

**Figure 13:**
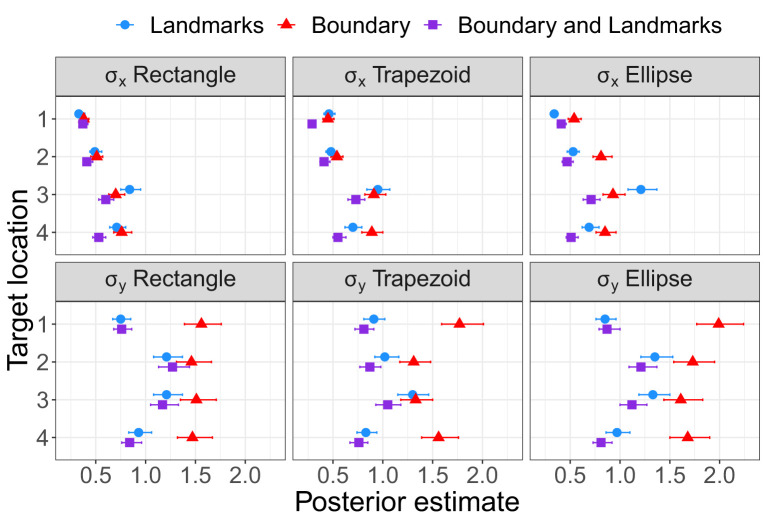
Modes and 95% HDI boundaries for parameter estimates of standard deviation on the x-axis and the forward y-axis in Experiment 2.

As for Experiment 1, Bayesian estimation was applied to quantify the effects of the presence of a boundary and/or landmarks on bias and precision. The modes and the lower and upper boundaries of 95% highest-density intervals of posterior distributions of parameter estimates are shown in Tables 4–6 for the intercepts β_x0_ and β_y0_ and rotation angles for the boundary groups Rectangle, Trapezoid, and Ellipse, respectively. Figures 12, 13 show modes and 95% HDI boundaries for the deflections created by Landmarks, Boundary, and Boundary-and-Landmarks β_x_[i] and β_y_[i] and standard deviations σ_x_[i] and σ_y_[i], respectively.

**Table 4:** Parameter estimates of intercepts for bias on the x-axis and on the forward y-axis and rotation angles of response point clouds for the Rectangle group in Experiment 2.

	**PSRF**	**ESS**	**Mode**	**HDI low**	**HDI high**
**Target 1**					
bx0 intercept	1.00	129,707	−0.11	−0.14	−0.07
by0 intercept	1.00	38,163	−0.63	−0.74	−0.53
θ Landmarks	1.00	88,772	5.51	0.19	10.47
θ Boundary	1.00	76,646	4.68	2.36	7.00
θ Boundary and Landmarks	1.00	90,754	5.80	−0.25	11.64
**Target 2**					
bx0 intercept	1.00	85,776	−0.23	−0.27	−0.18
by0 intercept	1.00	84,138	−0.92	−1.05	−0.80
θ Landmarks	1.00	70,924	15.41	12.26	18.51
θ Boundary	1.00	66,306	12.50	9.64	15.17
θ Boundary and Landmarks	1.00	65,058	12.96	10.75	15.43
**Target 3**					
bx0 intercept	1.00	61,914	−0.51	−0.58	−0.44
by0 intercept	1.00	55,332	−0.74	−0.87	−0.62
θ Landmarks	1.00	83,519	22.84	14.26	31.20
θ Boundary	1.00	82,322	21.16	18.62	23.94
θ Boundary and Landmarks	1.00	77,774	21.74	18.06	25.19
**Target 4**					
bx0 intercept	1.00	125,052	−0.40	−0.46	−0.33
by0 intercept	1.00	112,649	−0.46	−0.57	−0.35
θ Landmarks	1.00	89,655	29.64	21.03	37.64
θ Boundary	1.00	72,447	18.18	13.47	22.77
θ Boundary and Landmarks	1.00	79,136	18.39	11.28	26.08

PSRF, Potential Scale Reduction Factor; ESS, Effective Sample Size; HDI low and HDI high, Lower and upper boundaries of 95% highest density intervals.

**Table 5:** Parameter estimates of intercepts for bias on the x-axis and on the forward y-axis and rotation angles of response point clouds for the Trapezoid group in Experiment 2.

	**PSRF**	**ESS**	**Mode**	**HDI low**	**HDI high**
**Target 1**					
bx0 intercept	1.00	107,469	−0.13	−0.17	−0.09
by0 intercept	1.00	40,146	−0.38	−0.50	−0.27
θ Landmarks	1.00	76,234	12.90	7.24	18.28
θ Boundary	1.00	94,370	1.84	−0.61	4.55
θ Boundary and Landmarks	1.00	76,666	7.32	3.93	11.11
**Target 2**					
bx0 intercept	1.00	96,433	−0.29	−0.34	−0.25
by0 intercept	1.00	94,510	−0.50	−0.61	−0.40
θ Landmarks	1.00	70,934	16.42	12.20	20.34
θ Boundary	1.00	70,951	11.34	7.65	15.42
θ Boundary and Landmarks	1.00	73,510	15.70	11.17	20.03
**Target 3**					
bx0 intercept	1.00	127,495	−0.46	−0.54	−0.37
by0 intercept	1.00	134,675	−0.76	−0.88	−0.64
θ Landmarks	1.00	87,594	17.28	4.29	29.46
θ Boundary	1.00	84,575	13.07	1.63	24.44
θ Boundary and Landmarks	1.00	79,412	22.14	13.10	30.70
**Target 4**					
bx0 intercept	1.00	128,074	0.001	−0.07	0.07
by0 intercept	1.00	89,107	−0.33	−0.44	−0.23
θ Landmarks	1.00	90,148	28.00	11.31	44.16
θ Boundary	1.00	77,354	17.69	11.69	23.75
θ Boundary and Landmarks	1.00	84,408	15.09	2.10	29.09

PSRF, Potential Scale Reduction Factor; ESS, Effective Sample Size; HDI low and HDI high, Lower and upper boundaries of 95% highest density intervals.

**Table 6:** Parameter estimates of intercepts for bias on the x-axis and on the forward y-axis and rotation angles of response point clouds for the Ellipse group in Experiment 2.

	**PSRF**	**ESS**	**Mode**	**HDI low**	**HDI high**
**Target 1**					
bx0 intercept	1.00	120,263	−0.04	−0.08	0.0009
by0 intercept	1.00	53,061	−0.98	−1.11	−0.85
θ Landmarks	1.00	91,138	−1.89	−6.24	2.73
θ Boundary	1.00	89,947	1.05	−1.68	3.93
θ Boundary and Landmarks	1.00	78,320	11.06	6.35	16.03
**Target 2**					
bx0 intercept	1.00	93,173	−0.20	−0.26	−0.14
by0 intercept	1.00	107,243	−1.17	−1.31	−1.03
θ Landmarks	1.00	76,235	9.23	5.24	13.01
θ Boundary	1.00	77,108	9.24	3.43	14.23
θ Boundary and Landmarks	1.00	70,574	12.59	9.20	15.84
**Target 3**					
bx0 intercept	1.00	89,846	−0.72	−0.81	−0.63
by0 intercept	1.00	112,398	−1.28	−1.41	−1.15
θ Landmarks	1.00	108,221	40.59	33.57	47.04
θ Boundary	1.00	84,061	11.85	4.68	19.48
θ Boundary and Landmarks	1.00	78,781	22.37	16.09	28.62
**Target 4**					
bx0 intercept	1.00	135,736	−0.17	−0.24	−0.10
by0 intercept	1.00	106,088	−0.53	−0.64	−0.41
θ Landmarks	1.00	80,187	24.78	16.19	33.66
θ Boundary	1.00	78,341	11.48	5.81	17.29
θ Boundary and Landmarks	1.00	82,841	17.01	8.57	24.63

PSRF, Potential Scale Reduction Factor; ESS, Effective Sample Size; HDI low and HDI high, Lower and upper boundaries of 95% highest density intervals.

Higher underestimation of distances on the forward axis with increasing distance of target locations is indicated by more negative estimates for β_y0_ for the more distant target locations 2 and 3 than for target locations 1 and 4, for instance, −0.92 and −0.74 vs. −0.63 and −0.46, respectively, for the Rectangle group (Table 4, and similarly for Trapezoid and Ellipse in Tables 5, 6). The inward bias indicated by more negative values of β_x0_ was most pronounced for the most lateral target location 3 and in the Rectangle group also for the equally lateral target location 4 (Tables 4–6).

The presence of a boundary without landmarks resulted in a relative inward bias as indicated by negative deflections β_x_ (Figure 12) particularly for target locations closer to the boundary (not for target location 1), in contrast, the presence of landmarks without a boundary in the chosen configuration resulted in a reduced inward bias and thus a relative outward bias as indicated by positive deflections β_x_ (not for target location 1). On the forward axis, the presence of a boundary without landmarks resulted in a relatively strong distance underestimation as indicated by negative deflections β_y_, particularly for target location 4 closest to the participant on the forward axis. In contrast, the presence of landmarks in the chosen configuration reduced the underestimation of distance on the forward axis (not for target location 1).

Most clearly for precision on the forward axis as indicated by low standard deviations σ_y_ in Figure 13, Landmarks increased precision in the chosen configuration compared to Boundary only, and the combination of Boundary-and-Landmarks increased precision further only slightly if at all. For precision on the x-axis, the differences between environment conditions were less pronounced and less consistent across target locations, however, increased precision with the combination of Boundary-and-Landmarks compared to Landmarks or Boundary only was observed for target locations 3 and 4.

Rotation angles θ in Tables 4–6 reflect an alignment of response distributions with lateral boundaries particularly for target location 4 with lower rotation angles for Boundary than Landmarks. The same difference is apparent in rotation angles for target location 3 in the Trapezoid and Ellipse groups.

### 4.3. Discussion

Landmarks alone and in combination with a boundary did reduce absolute distance error in reproducing locations compared to the Boundary condition. However, real walking that provided idiothetic in addition to visual cues to self-motion did not reduce distance error compared to passive translation. Either idiothetic cues provide no advantage in spatial updating across forward self-motion for target locations that remain in vista space or visual cues provided by spatial context took primacy in the present experiment. It is possible that an effect of idiothetic cues could be shown if static visual reference would be eliminated (e.g., by showing only flaring random dots as floor texture as in Wolbers et al., 2008). Yet, some static visual reference is typically available in VR and teleoperation scenarios.

Landmarks were located inside the boundary and the rectangle and trapezoid boundaries contained vertices that could function as landmarks. In addition to the absence of vertices, the area inside the boundary stretched further ahead for the ellipse whereas it was limited by a wall perpendicular to the forward axis for rectangle and trapezoid. Vertices and the limited stretch presumably are the reasons why, in the boundary-only condition, the distance error was smaller for the rectangle and trapezoid than for the ellipse. Such an advantage of the Rectangle and Trapezoid groups could also explain smaller distance error than in the Ellipse group in the landmarks-only condition with the additional assumption that the (imagined) wall ahead took effect even in trials in which it was not shown. An alternative explanation would be that the average performance of participants in the Ellipse group was lower because of interindividual differences. As in Experiment 1, the general underestimation of distances in VR was confirmed again, but landmarks reduced distance underestimation.

The presence of landmarks inside the boundary reduced distance error by reducing bias and increasing precision. The boundary pushed responses inside for targets close to the boundary consistent with similar results in Negen et al. (2020), and landmarks when present counteracted this bias. For locations enclosed between a boundary and an inside landmark, cue combination increased precision. For location 1 which was enclosed between inside landmarks but more distant from the boundary, the cue combination seems restricted to the two landmarks because the additional presence of a boundary did not increase precision further.

The orientation of walls mattered. In the Boundary condition, the response distributions were more aligned with the lateral boundary. Laterally, the boundaries were closer to lateral target locations than in Experiment 1, however, there were no inside landmarks in Experiment 1 and hence, we cannot be sure that this alignment with a lateral boundary would be as strong if a comparison condition contained no inside landmarks.

In summary, we succeeded in supporting forward updating by landmarks inside boundaries and demonstrated the benefits of a boundary wall perpendicular to the motion direction as well as of static visual cues enclosing target locations (decreased bias and increased precision). Visual cues took primacy over idiothetic cues for pure forward updating in vista space in immersive VR.

## 5. General discussion

We were interested in factors contributing to spatial updating of location memory across forward self-motion in VR. Increased optic flow (overhead sleepers during translation) provided no clear advantage with passive forward translation in Experiment 1 and no advantage was obtained for real walking compared to passive translation in Experiment 2. The missing advantage for real walking and the small Sleepers effect even without any wall is consistent with the presumption that very little dynamic visual stimulation aka optic flow is sufficient for continuous forward updating and thus, passive forward translation experienced in an MRI scanner may generate brain activation similar to real translation scenarios (e.g., Wolbers et al., 2008). Yet, to be sure that idiothetic cues provide no advantage for forward updating as soon as the minimal optic flow is provided, the passive translation would need to be compared with real walking in minimal optic flow conditions (e.g., flaring random dots). Note, however, that idiothetic cues (real walking) for translation are critical for establishing a cognitive map during and for navigation (Ruddle and Lessels, 2006; Ruddle et al., 2011).

The richness of spatial context and proximity of landmarks to targets improved updating. We found supporting effects of static visual cues in both experiments. A lateral boundary with distinctive features when close (Experiment 1), a boundary perpendicular to the forward axis (Experiment 2), and especially close landmarks inside boundaries (Experiment 2) reduced biases and increased precision in reproducing target locations. The combined effect of landmarks and boundaries (cue combination, Newman and McNamara, 2022) was particularly strong if targets were enclosed between landmarks and boundaries. Thus, for forward updating in vista space in immersive VR, the close visual spatial reference seems to be the most promising means of support.

If self-motion would have entailed rotation of more than say 90°, real walking may well have provided support by idiothetic cues to self-motion (e.g., Gramann et al., 2021) because then, changes in head direction need to be accounted for in updating mechanisms as specified in current neuroscientific theories (Byrne et al., 2007; Bicanski and Burgess, 2018). However, the effects of real walking with rotation in updating are presumably stronger in sparse spatial contexts or may even be restricted to sparse spatial contexts (Riecke et al., 2007). The present results let us expect that close visual cues as a spatial reference would take primacy over idiothetic cues even with rotation. Boundary shapes and corners as boundary features had smaller effects than inside landmarks in the present experiments. If self-motion entails rotation, however, features of boundary shapes become important that can provide cues to orientation in the environment, for instance, those with corners as opposed to no corners, or lower as opposed to higher degrees of rotational symmetry (e.g., trapezoid vs. circle). Similarly, distant landmarks beyond the boundary are important orientation cues across extensive rotation which can be added as AR support to an egocentric view (e.g., Liu et al., 2022).

Restricting self-motion to forward translation or forward walking allowed us to reliably determine biases in the x- and y-directions in reproducing locations and how these biases were influenced by boundaries and landmarks, more reliably than would have been possible if participants had experienced variable rotation across trials. Presumably, participants worked with both egocentric and allocentric spatial representations including egocentric representations in the fronto-parietal network underlying spatial working memory (Curtis, 2006; Kesner and Creem-Regehr, 2013) and spatial representations (allocentric and egocentric) in parahippocampal and hippocampal cortex (Byrne et al., 2007; Bicanski and Burgess, 2018). Only requiring forward updating in vista space kept egocentric and allocentric frames aligned and did not pose the challenge of transforming between allocentric and egocentric representations (which reliably involves retrosplenial cortex; Gramann et al., 2021). It is noteworthy that according to recent findings employing single-cell recording in humans (Kunz et al., 2021), continuous updating of egocentric representations in the parahippocampal cortex across self-motion that included rotation (inferred from firing rates of egocentric bearing cells) occurred based on purely visual information in non-immersive VR (laptop screen). Thus, our results suggesting visual primacy obtained with an HMD may generalize to less immersive VR with scenes that provide at least some static visual reference. Static visual spatial reference if available seems to dominate visual and idiothetic dynamic cues for updating location memory even though in immersive VR optic flow stimulates over a large field of view. However, static and dynamic visual cues have not been discerned in the present paradigm. Optic flow is also available in sparse environments and even more so if static visual cues are present. Thus, any static visual cues also contribute to dynamic visual cues for updating.

For supporting location memory in VR, close (inside) landmarks as spatial reference have proven more effective than boundaries. This may also be true for supporting spatial updating in VR as assessed by triangle completion. It is straightforward to include rotation in experienced self-motion and to test the effect of close landmarks on reproducing locations or triangle completion (reproducing the starting location) as in Sjolund et al. (2018), for example. Another interesting follow-up to the present experiments would be to employ conditions introducing inconsistencies between boundary and landmarks, for instance, rotating the landmark configuration inside the boundary between encoding and reproducing locations or during the outward path in triangle completion. Such manipulations allow us to study the weighing of cues in cue combination and whether available cues are combined optimally given their reliabilities. A cue combination of visual and idiothetic cues has been studied before for triangle completion in sparse environments (e.g., Chen et al., 2017; Sjolund et al., 2018).

The scenes in Experiment 1 were sparse and the shape of the lateral wall changed from trial to trial while the forward pair of poles remained the same. The participant perspective was abruptly reset to the starting location after translation trials. In contrast, in Experiment 2, the scene remained unchanged within a block of trials and the participant perspective changed continuously. Thus, in Experiment 2, conditions were more favorable for establishing a scene representation, however, we presume that the constancy of the geometric layout across trials in Experiment 1 also induced the experience of a single scene or at least of scenes with a constant layout. In Experiment 2, the landmark conditions were equivalent across the boundary groups, still, there was a slight advantage of the rectangle and Trapezoid groups in landmark conditions. Hence, if the Ellipse group was not a group containing individuals performing generally lower, this advantage can be explained by carry-over effects from earlier blocks with boundaries. Participants may have imagined the boundary that they had experienced in trials with a boundary in later landmark trials.

The landmark objects in Experiment 2 were placed to indicate the forward direction (chair) and to provide spatial reference in variable distance to target locations (plant, ball). Landmarks to the left had symmetric counterparts to the right. Thus, the landmarks formed a regular configuration, a polygon symmetric at the forward axis. The lines connecting symmetric landmarks were perpendicular to the forward axis. In Experiment 2, targets were never located on these lines, but if they had been, bias on the y-axis probably had been reduced to almost zero for these trials. Similarly, strong support by spatial reference can be expected for other salient locations in landmark configurations (e.g., the center). Furthermore, configurations as boundary shapes can entail prominent axes that contribute to allocentric reference. Effects of such allocentric reference (supporting and distorting) have been studied extensively in research on spatial memory (e.g., Shelton and McNamara, 2001; Zhou and Mou, 2019). Hence, if local landmarks are added to scenes to support spatial memory, orientation, and navigation, the effects of configurations of landmarks (Newman and McNamara, 2022) and configurations with boundary features can be considered for amplifying their utility.

In the present study, we have demonstrated the primacy of static visual cues in spatial updating across forward translation in immersive VR for reproducing target locations in vista space by quantifying in detail reductions in bias and increases in precision. Landmarks combined with boundaries provide spatial context that according to behavioral and neuroscientific evidence provides a reference frame that is used to establish egocentric and allocentric spatial representations. Allocentric representations contain the ego-location in the present scene and are employed in updating egocentric representations. We studied close landmarks inside boundaries and based on our results suggest to strongly consider providing not only distant (Liu et al., 2022) but also close landmarks in virtual environments and as augmented reality features (Suzuki et al., 2022) in synthetic environments (e.g., in teleoperation) for alleviating challenges to spatial orientation resulting from reduced sensory feedback about self-motion.

## Data availability statement

The datasets presented in this study can be found at: https://osf.io/e39jf/.

## Ethics statement

The studies involving human participants were reviewed and approved by the Ethics Committee of the Faculty of Behavioral and Social Sciences of Chemnitz University of Technology. The patients/participants provided their written informed consent to participate in this study.

## Author contributions

SW set up the experiments in Unity. ZB and JB conducted the experiments. ZB and GJ performed the statistical analyses and wrote sections of the manuscript. All authors contributed to the conception of the experiments, read, and approved the submitted version.
