# Supplementary material for: Spatial updating in virtual reality for reproducing object locations in vista space—Boundaries, landmarks, and idiothetic cues
Source: Front Psychol. 2023 Jun 22;14:1144861. doi: 10.3389/fpsyg.2023.1144861 (PMC10325663; doi:10.3389/fpsyg.2023.1144861)
Supplement: Supplementary file 1 [file Data_Sheet_1.docx]

Supplementary Material

Spatial Updating in Virtual Reality for Reproducing Object Locations in Vista Space – Boundaries, Landmarks, and Idiothetic Cues

Zhanna Borodaeva, Sven Winkler, Jennifer Brade, Philipp Klimant, Georg Jahn*

*** Correspondence:** Georg Jahn: georg.jahn@psychologie.tu-chemnitz.de

# Supplementary Figures

**Supplementary Figure 1.** Mean response coordinates with ellipses capturing about 80% of responses in Translation trials Experiment 1 showing the data in Figure 3 separately for left and right target locations.

**Supplementary Figure 2.** Mean response coordinates with ellipses capturing about 80% of responses in No Translation trials Experiment 1.

**Supplementary Figure 3.** Mean response coordinates with ellipses capturing about 80% of responses for the Rectangle group in Experiment 2 showing the data in Figure 9 separately for left and right target locations.

**Supplementary Figure 4.** Mean response coordinates with ellipses capturing about 80% of responses for the Trapezoid group in Experiment 2 showing the data in Figure 10 separately for left and right target locations.

**Supplementary Figure 5.** Mean response coordinates with ellipses capturing about 80% of responses for the Ellipse group in Experiment 2 showing the data in Figure 11 separately for left and right target locations.
